# Cytokine, Antibody and Proliferative Cellular Responses Elicited by *Taenia solium* Calreticulin upon Experimental Infection in Hamsters

**DOI:** 10.1371/journal.pone.0121321

**Published:** 2015-03-26

**Authors:** Fela Mendlovic, Mayra Cruz-Rivera, Guillermina Ávila, Gilberto Vaughan, Ana Flisser

**Affiliations:** 1 Departamento de Microbiología y Parasitología, Facultad de Medicina, Universidad Nacional Autónoma de México, Av. Universidad 3000, Coyoacán, México D.F. 04510, México; 2 Facultad de Ciencias de la Salud, Universidad Anáhuac, México Norte, Av. Universidad Anáhuac 46, Huixquilucan, 52786 Edo. de México, México; 3 AV BioSciences, Mexico City, Mexico; Instituto de Diagnostico y Referencia Epidemiologicos, MEXICO

## Abstract

*Taenia solium* causes two diseases in humans, cysticercosis and taeniosis. Tapeworm carriers are the main risk factor for neurocysticercosis. Limited information is available about the immune response elicited by the adult parasite, particularly the induction of Th2 responses, frequently associated to helminth infections. Calreticulin is a ubiquitous, multifunctional protein involved in cellular calcium homeostasis, which has been suggested to play a role in the regulation of immune responses. In this work, we assessed the effect of recombinant *T*. *solium* calreticulin (rTsCRT) on the cytokine, humoral and cellular responses upon experimental infection in Syrian Golden hamsters (*Mesocricetus auratus*). Animals were infected with *T*. *solium* cysticerci and euthanized at different times after infection. Specific serum antibodies, proliferative responses in mesenteric lymph nodes and spleen cells, as well as cytokines messenger RNA (mRNA) were analyzed. The results showed that one third of the infected animals elicited anti-rTsCRT IgG antibodies. Interestingly, mesenteric lymph node (MLN) cells from either infected or non-infected animals did not proliferate upon *in vitro* stimulation with rTsCRT. Additionally, stimulation with a tapeworm crude extract resulted in increased expression of IL-4 and IL-5 mRNA. Upon stimulation, rTsCRT increased the expression levels of IL-10 in spleen and MLN cells from uninfected and infected hamsters. The results showed that rTsCRT favors a Th2-biased immune response characterized by the induction of IL-10 in mucosal and systemic lymphoid organs. Here we provide the first data on the cytokine, antibody and cellular responses to rTsCRT upon *in vitro* stimulation during taeniasis.

## Introduction

*Taenia solium* is responsible for two diseases in humans, i.e. taeniosis and neurocysticercosis, which are caused by the adult tapeworm and the larval stage (cysticercus), respectively. Tapeworms lodge in the small intestine of human beings after ingestion of live cysticerci in contaminated, undercooked pork meat; develop into adult tapeworms and expel gravid proglottids full of eggs in feces. Accidental intake of eggs by humans results in the development of neurocysticercosis, due to the establishment of cysticerci in the central nervous system [1]. Neurocysticercosis is a public health problem in many developing countries [2]. Taeniosis is usually asymptomatic but epidemiological studies have shown that tapeworm carriers are the main risk factor for developing neurocysticercosis [3]. Human beings are the only definitive hosts for *T*. *solium*. Thus, the use of experimental models, such as the Syrian golden hamster (*Mesocricetus auratus*), is necessary to study the mechanisms involved in the immune response elicited by the tapeworm [4].

Helminth infections generally induce Th2 responses. Molecules derived from helminths that stimulate Th2 responses have been subject of current research for their potential regulatory immune functions [5,6]. However, only few molecules involved in triggering such responses have been recognized [7]. Calreticulin (CRT) is a ubiquitous and well-conserved protein found in all living cells except prokaryotes and fungi. CRT is a multifunctional protein with a housekeeping role in cellular calcium homeostasis and glycoprotein synthesis [8,9]. Additionally, there is growing evidence supporting its role in the induction and regulation of immune responses in different parasitic diseases [10]. For example, *Schistosoma mansoni* CRT is a strong T-cell immunogen capable of inducing IL-4 synthesis [11], while native *Heligmosomoides polygyrus* CRT has been shown to induce production of IL-4 and IL-10 by T cells from infected mice [12], suggesting that CRT is able to induce a Th2 response during helminth infections.

We have previously identified, cloned and expressed *T*. *solium* CRT as a recombinant protein (rTsCRT) with calcium binding functions and analyzed its expression pattern during *T*. *solium* development [12,13]. We have also characterized the immune response elicited by rTsCRT as an oral vaccine [14–16]. Nonetheless, the immune response to rTsCRT upon tapeworm infection has not been investigated. The aim of the present study was to characterize the cytokine, humoral and cellular immune responses against rTsCRT during experimental infection in the hamster model.

## Methods

### Ethics Statement

The Institutional Research and Ethics Committee of the Faculty of Medicine, National University of Mexico (UNAM) in accordance with the Mexican Official Guidelines (NOM-062-ZOO-1999) approved all animal procedures and the experimental protocol (Approval numbers for immune response experiments: 004–2010 and quantitative RT-PCR: 020–2011).

### *T*. *solium* infection

Six-month-old outbreed female Syrian hamsters (*Mesocricetus auratus*) were supplied with water and food *ad libitum*. Five animals were kept *per* cage and light/darkness was on a 12:12 hour cycle. Two weeks prior to infection, all hamsters were treated for intestinal parasites with three daily doses of albendazole (30mg/Kg) and a single dose of praziquantel (30 mg/kg). Two experiments were performed and hamsters were infected orally with 4 *T*. *solium* cysticerci obtained from the skeletal muscle of one naturally infected pig per experiment. Prior to infection, cysticerci were assayed for viability by *in vitro* evagination in the presence of 25% porcine bile and only cysticerci from pigs that had parasites with >90% evagination were used. All hamsters in each experiment were infected at day 1 and were euthanized at 10, 20, and 30 days post infection (DPI) by inhalation of sevofluorane (Svofast, Baxter Int., Deerfield, IL) Two control groups were left uninfected. Blood, spleen and mesenteric lymph node (MLN) cells were collected for ELISA, lymphocyte proliferation assays and RT-PCR analyses.

### Preparation of tapeworm crude extract and excretion/secretion products

Excretion and secretion (E/S) products and crude extract (TsCE) from tapeworms were prepared as follows: Hamsters were orally infected with 8 cysticerci at day 1 and immunosuppressed with methyl prednisolone acetate (2 mg, Depo-medrol, Pfizer, Mexico) at days 1,15 and 30 [17]. *T*. *solium* mature tapeworms were obtained at 35 DPI, rinsed and incubated in sterile RPMI supplemented with antibiotics (Gibco, Grand Island, NY) for 24h at 37°C. For E/S products preparation culture medium was centrifuged at 3300xg for 15 min and E/S products were concentrated 100 fold using a Millipore concentrator (Millipore Corp, Bedford, MA) with a 10kDa molecular weight cut-off. For TsCE preparation, tapeworms were recovered from the culture medium, washed with phosphate buffer saline (PBS) and homogenized in 6.7 mM phosphate buffer plus 0.4 mM KCl and 1 mM MgCl_2_, pH 7.4 using a homogenizer (PowerGen125, Fisher Scientific, UK). Homogenates were then sonicated 3 times (Omniruptor 250, Omni Int. Inc., GA), centrifuged at 12000xg for 30 min to eliminate any particulate material and filtered using a 0.2 μm membrane. All working solutions contained complete protease inhibitors (Roche Applied Science, Indianapolis, IN). Bradford protein assay (Bio-Rad, Hercules, CA) was used to measure protein concentration. Aliquots were kept at -70°C until use.

### Expression and purification of recombinant TsCRT

The full coding region of the mature rTsCRT was cloned, expressed and the resulting protein was purified as described previously [13,14]. Briefly, *Escherichia coli* bacterial cultures were induced to express rTsCRT, treated with 20% sucrose in Tris buffer, centrifuged and sonicated. rTsCRT was submitted to 10% SDS-PAGE, the 50kDa band was electro-eluted. Residual LPS was eliminated by Detoxi-gel endotoxin removing columns (Pierce Biotechnology, IL, USA) and endotoxins were measured as reported elsewhere [14]. Purified rTsCRT was filtered using a 0.2μm filter and kept at -70°C until use.

### Electrophoresis and western blot analysis of E/S products

To evaluate if TsCRT was secreted/excreted by the tapeworms, 25μg of E/S products were diluted in loading buffer under reducing conditions, boiled at 100°C for 5 min, and subjected to SDS-PAGE using a 10% polyacrylamide gel. Proteins were blotted to PVDF membranes (Millipore, Billerica, MA) and incubated with either anti rTsCRT or control sera at a 1:5000 dilution [12]. HRP-labeled goat anti mouse IgG was used as secondary antibody (Zymed, CA). Blots were developed using 3–3’ diaminobenzidine.

### Analysis of antibody detection by enzyme-linked immunosorbent assay

Serum samples were analyzed by ELISA for the specific detection of anti-rTSCRT and anti-TsCE IgG antibodies as described previously [18]. Biotin conjugated anti-hamster IgG (eBioscience, San Diego, CA), streptavidin-HRP and ABTS (Invitrogen) were used. All incubations were followed by three washings with PBS plus 0.05% Tween 20. Absorbance was measured at 405 nm. The cut-off value was defined as the mean of the optical density obtained with the serum from uninfected animals plus two standard deviations.

### Lymphocyte proliferation assay

Spleens and mesenteric lymph nodes (MLN) were dissected from uninfected and infected hamsters at 10, 20 and 30 DPI. Cell suspensions were obtained by disaggregation through a 70 μm cell strainer (BD Biosciences, San Jose, CA). 0.15 M ammonium-chloride-potassium buffer was used to lyse erythrocytes. The resulting cell suspensions were washed three times with RPMI culture medium with antibiotics (Gibco) by centrifugation at 1200 rpm at 4°C. Cells were counted, suspended in 10% FCS-supplemented RPMI and plated in 96-well flat-bottom plates (Nalge Nunc Int., Rochester, NY) in presence of medium alone, 10 μg/ml rTsCRT, 25 μg/ml TsCE or 2 μg/ml concanavalin A (Con A, Sigma-Aldrich, Saint Louis, MO). Triplicate cultures consisting of 2x10^5^ cells were maintained at 37°C in a CO_2_ incubator for 5 days. Eight hours before harvesting, ^3^H thymidine (PerkinElmer, Wellesley, MA) was added to each well and the radioactive label was measured using a cell harvester and counter (MicroBeta Trilux, PerkinElmer).

### RNA isolation and cytokine quantification by quantitative RT-PCR

Spleen and MLN cells were isolated and stimulated as described above. Triplicate cultures consisting of 3x10^5^ were maintained at 37°C in a CO_2_ incubator for 5 days, and plated in 96-well flat-bottom-plates. RNA was isolated using Trizol reagent (Invitrogen, Carlsbad, CA) following the manufacturer’s recommendations. RNA quality was assessed by agarose gel electrophoresis and quantified at 260 nm using a Genequant spectrophotometer (BioRad). RNA samples were treated with DNase (Invitrogen) following manufacturer’s instructions. cDNA was prepared from 1.2 μg RNA by reverse transcription using a Superscript First Strand Synthesis System (Invitrogen). The cDNA samples were kept at -20°C until use. TibMolbiol LLC (Adelphia, NJ) designed the primers for β-actin, IL-4 and IL-5; the primers for IL-10 were synthesized according to Espitia *et al*. [19]. Nucleotide sequences, Tm values, product size and Genebank accession numbers are shown in Table 1.

**Table 1:** Nucleotide sequences of primers for hamster genes.

Cytokine	Primer sequence	Tm (°C)	GenBank accesion number
**β-Actin F**	5' TGTACCCAGGCATTGCTGAC 3'	58.2	AF047041
**β-Actin R**	5' TCATCGTACTCCTGCTTGCTGA 3'	58.5	
**IL-4 F**	5' CTTCTAGCATGTACCGGGAACTG 3'	58.8	AF04621
**IL-4 R**	5' CTTCAAGCACAGGGTCACCTC 3'	58.8	
**IL-5 F**	5' GCCGTAGCCATGGAGATC 3'	54.6	JQ290352
**IL-5 R**	5' CGATGCACAGCTGGTGGTGAT 3'	55.4	
**IL-10 F**	5' GGTTGCCAAACCTTATCAGAAATG 3'	54.0	AF046210
**IL-10 R**	5' TTCACCTGTTCCACAGCCTTG 3'	54.4	

Tm: Melting temperature

For each primer combination, optimal primer and MgCl_2_ concentrations, as well as annealing temperatures were experimentally determined. cDNA amplification was performed using the LightCycler 2.0 instrument (Roche Applied Science). The reaction mixture consisted of 1μl cDNA, 3mM MgCl_2_, 0.5μM of each primer, and 1μl LightCycler FastStart DNA Master SYBR I Green mix (Roche Applied Science) in a final volume of 10μl. For the non-template control, water was added to the reaction instead of cDNA. The PCR cycling conditions were as follows: initial denaturation at 95°C for 10 min, followed by 45 cycles each consisting of denaturation at 95°C for 10s, annealing at 60°C for 5s, and extension at 72°C for 9s. Fluorescence was acquired after each extension phase. After amplification, a melting curve analysis was performed to assess the specificity of the product. Standard curves for each primer set were generated from ten-fold serial dilutions of cDNA starting at a 1:10 and used to calculate the PCR efficiencies using the LightCycler software. Relative quantification calculations were performed by the Pfaffl method [20]. Non-stimulated cells were used as controls in all experiment.

### Statistical Analysis

Data are shown as mean ± standard error of the mean (SEM). The Kruskal-Wallis test for non-parametric data with Dunn post-test was used. *P* values <0.05 were considered significant. Statistical analysis was performed using the Prism 6.0 software (Graphpad Prism, San Diego, CA).

## Results

### Presence of TsCRT in E/S of cultured tapeworms

**Fig 1:**
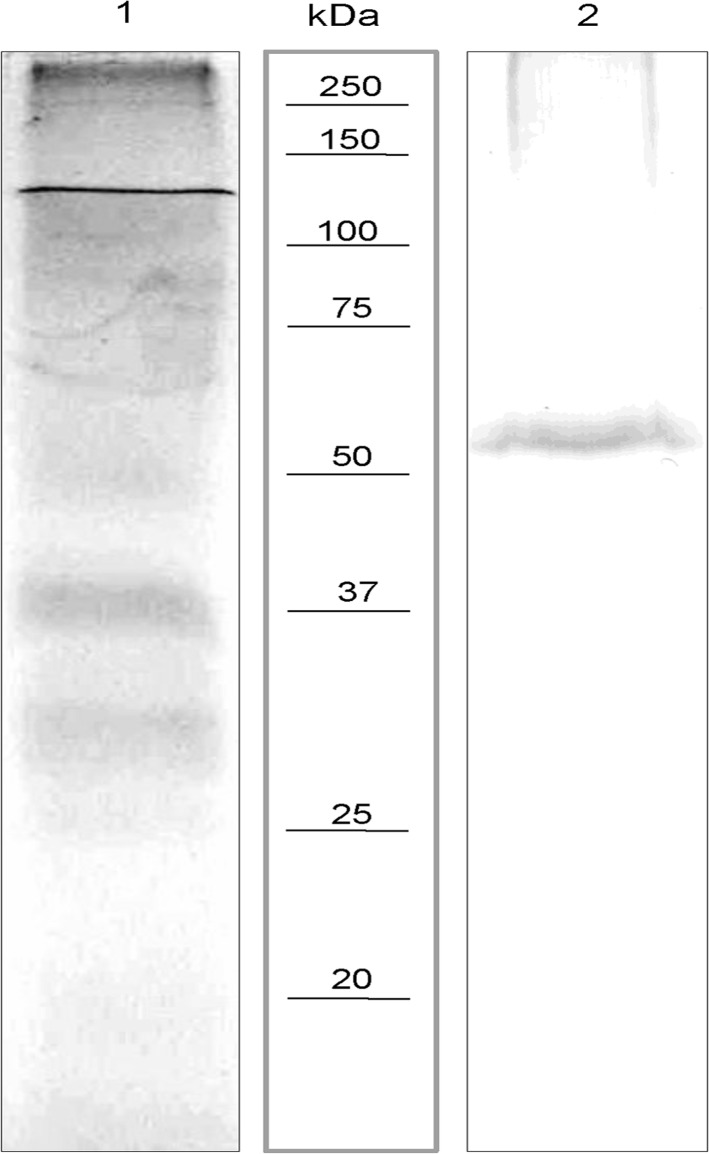
TsCRT identification in E/S products obtained from *in vitro* cultured tapeworms. E/S products were separated by SDS-PAGE and stained with Coomassie Brilliant Blue (lane 1). Samples were blotted to PVDF membranes after SDS PAGE and analyzed by western blot with anti-rTsCRT serum (lane 2). Molecular weight markers (kDa) are shown in the center lane.

The identification of TsCRT in E/S products was performed in supernatants of *in vitro* cultured tapeworms recovered from immunosuppressed hamsters and analyzed by western blot using specific anti-rTsCRT polyclonal antibodies. Among the proteins present in the *T*. *solium* E/S products separated by SDS-PAGE, a band of ~50 kDa reacted with the anti-rTsCRT serum (Fig. 1). This molecular weight corresponds to the expected size of TsCRT.

### TsCRT specific humoral immune response

**Fig 2:**
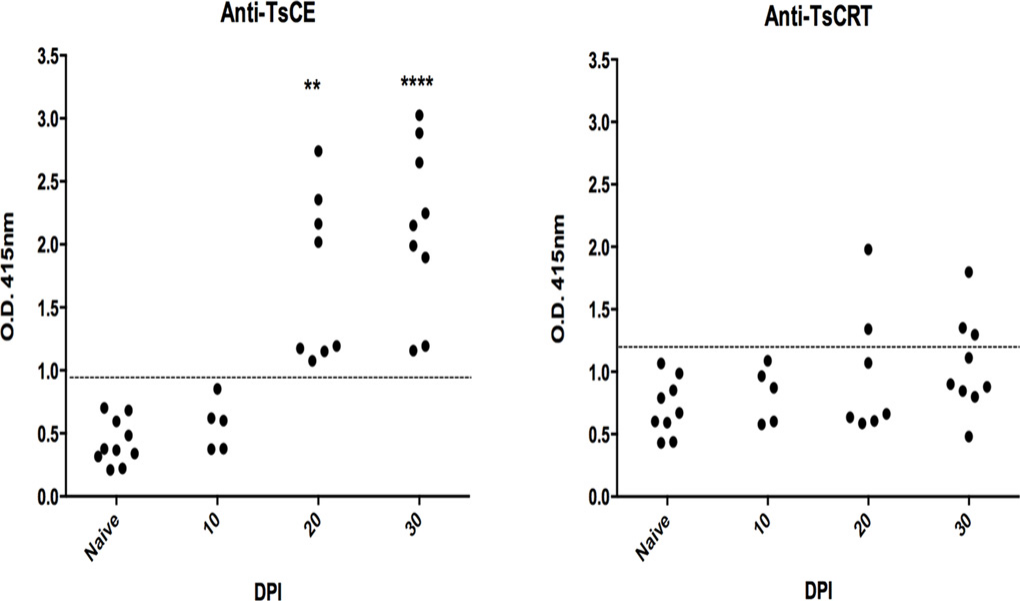
Presence of IgG antibodies after oral infection with *T*. *solium* cysticerci. ELISA testing was used to detect specific antibodies. A) Anti-TsCE IgG and B) anti-rTsCRT antibodies present in the sera obtained from uninfected and infected hamsters. Each dot refers to an individual hamster and the discontinuous lines represent the cutoff value that was calculated as the mean OD of the uninfected group plus 2 standard deviations. Kruskal-Wallis: ** p<0.01 and **** p<0.0001 as compared to the uninfected animals.

The capacity of TsCRT to induce a specific IgG response in infected animals at different DPI by ELISA was investigated. Fig. 2 shows the presence of specific IgG antibodies reacting to TsCE (Fig. 2A) and the purified rTsCRT (Fig. 2B). Between 28 and 33% of hamsters showed anti-TsCRT specific antibodies at 20 and 30 DPI respectively. Anti-TsCE OD values increased along the experiment starting at 10 DPI, and at 20 and 30 DPI all infected hamsters were positive (p<0.05).

### Local and systemic lymphoproliferative responses

**Fig 3:**
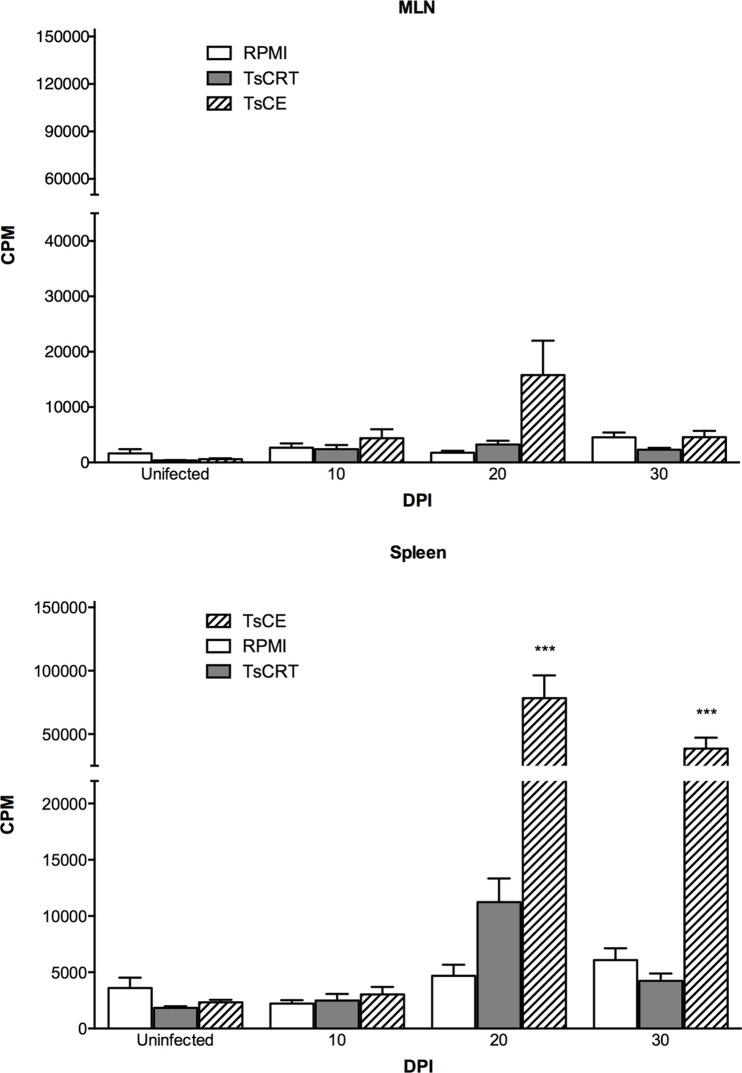
Proliferative responses of MLN (A) and spleen (B) cells from uninfected and infected hamsters to purified rTsCRT and TsCE. White bars represent data from cells incubated in RPMI alone, grey bars from cells incubated with rTsCRT, and hatched bars indicate the response to TsCE. Bars represent mean ± SEM of ^3^H thymidine incorporation from triplicate cultures of cells obtained from 6 animals. Kruskal-Wallis: *** P<0.001 as compared to the RPMI control.

The ability of TsCE and rTsCRT to induce *in vitro* responses during *T*. *solium* infection was assessed by lymphoproliferative assays in MLN (local) and spleen (systemic) cells. Con A was used as positive control for all proliferation assays (data not shown). Fig. 3 shows the proliferative response to TsCE and rTsCRT before infection and at different DPI. MLN cells did not show significant levels of proliferation in response to rTsCRT or TsCE (Fig. 3A). In contrast, TsCE induced a significant proliferative response in spleen cells at 20 and 30 DPI (P<0.05). rTsCRT showed an increase in the proliferation levels of spleen cells at 20 DPI that were not statistical significant (Fig. 3B).

### Cytokine profile induced during *T*. *solium* taeniosis

**Fig 4:**
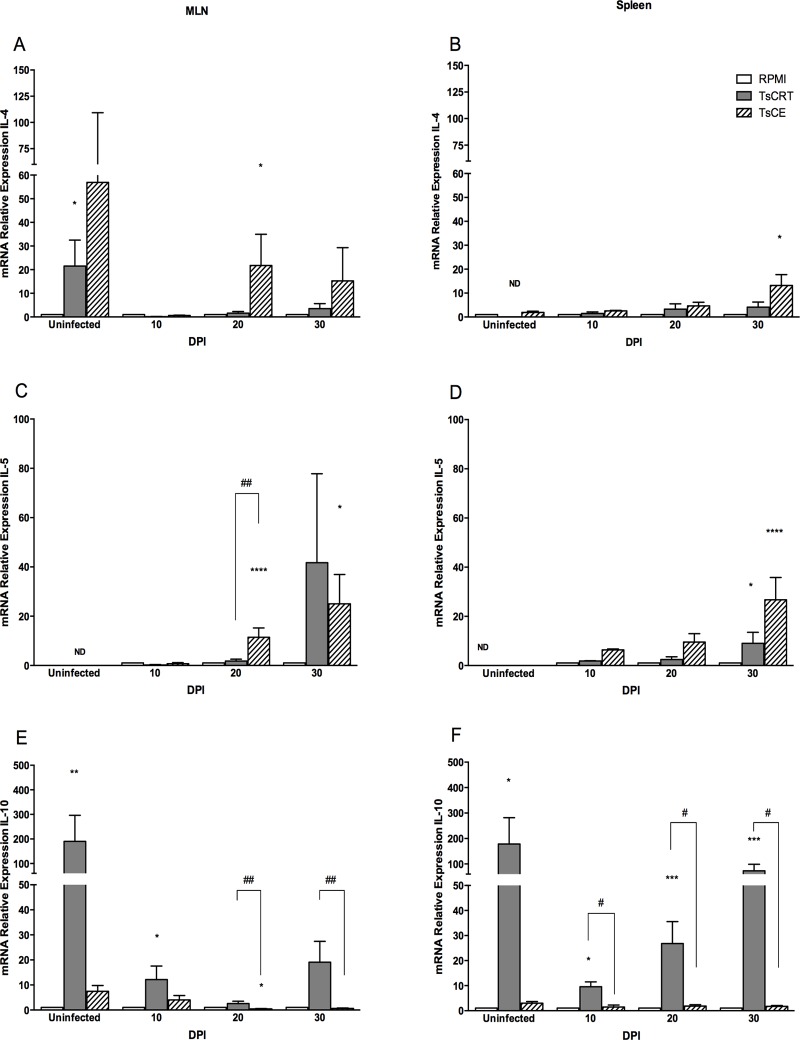
Cytokine mRNA expression by MLN and spleen cells after *in vitro* stimulation with TsCE and rTsCRT. cDNA was prepared from mRNA obtained from MLN (A, C, D) or spleen (B, D, E). mRNA levels of IL-4 (A, B) IL-5 (C, D) and IL-10 (E, F) were determined by quantitative RT-PCR. Data are expressed as the mean of the ratio of each cytokine relative to β-actin (housekeeping gene). White bars represent data from cells incubated in RPMI alone, grey bars from cells incubated with rTsCRT and hatched bars with TsCE. Bars represent mean ± SEM of relative expression from triplicate cultures of cells obtained from 6 animals. Kruskal-Wallis: * P<0.05, ** P<0.01, *** P<0.001, **** P<0.0001 rTsCRT-induced expression as compared to the RPMI control. ^#^ P<0.05, ^##^ P<0.01 rTsCRT-induced expression as compared to TsCE. ND, not detected.

To analyze if TsCE and rTsCRT induce a Th2 immune response during *T*. *solium* infection, the relative expression of IL-4, IL-5 and IL-10 in spleen and MLN cells from uninfected and infected hamsters in response to *in vitro* stimulation at different DPI was evaluated. Fig. 4 shows the expression of mRNA for IL-4, IL-5 and IL-10 relative to β-actin assessed by quantitative RT-PCR. Stimulation with TsCE resulted in an increased expression of IL-4 and IL-5 transcripts in both MLN and spleen cells at 20 and 30 DPI compared to non-stimulated cells (Fig. 4A, B, C, and D) while rTsCRT induced a significant increase of the IL-4 mRNA levels in MLN cells from uninfected hamsters (p<0.05) but not from spleen cells (Fig. 4A and B). Only MLN cells from infected hamsters at 30 DPI increased IL-5 mRNA expression in response to rTsCRT stimulation (P<0.05, Fig. 4D). Interestingly, cells from uninfected and infected hamsters significantly induced IL-10 mRNA expression in response to rTsCRT stimulation, with the exception of MLN cells from 20 DPI. Stimulation with TsCE did not result in a significant increase of transcript levels for the IL-10 immunoregulatory cytokine neither in MLN nor spleen cells (Fig. 4E and F).

## Discussion

Here we have described the immune response elicited by *T*. *solium* TsCRT upon infection in the hamster model. Interestingly, *T*. *solium* TsCRT induced a robust IL-10 response in both MLN and spleen cells from infected and uninfected animals within a prevalent Th2 microenvironment characterized by increased levels of IL-4 and IL-5. These findings are of importance owed to the fact that Th2 responses prevail during helminth infections. Additionally, the presence of native TsCRT in tapeworms E/S products was confirmed.

CRT is involved in cellular Ca2+ homeostasis; however, there is growing evidence of its role in immune regulation [10]. IL-10 expression induced by rTsCRT in local and systemic lymphoid organs upon *in vitro* stimulation is in agreement with previous reports [21]. This is important for the parasite life cycle as a mechanism to modulate the host immune response [22,23]. IL-10 induction is a common feature shared by different helminths, presumably, associated with parasite survival [24,25]. In human neurocysticercosis, asymptomatic patients usually present elevated levels of IL-10, suggesting that IL-10 might play a role in disease outcome [26]. Increased IL-10 production has been associated to immunoregulation in helminth chronic infection in humans and animal models [23], and also contribute to the maintenance of the Th2 response by inhibiting Th1 responses [27]. Additionally, IL-10 acts as an anti-inflammatory cytokine preventing the pathology associated with parasite infections, down-regulating the host immune response [6,28]. Thus, TsCRT might contribute to the overall masking strategy developed by *T*. *solium* to escape the host response in an unfavorable microenvironment, characterized by mediators and cytokines that influence parasite expulsion.

As observed in other helminths [29–31], Th2 type responses are primarily developed during *T*. *solium* infection. Several reports have suggested the pivotal role of IL-4 in host protective responses against parasite infections [32]. Interestingly, stimulation with TsCE, but not rTsCRT, resulted in increased synthesis of both IL-4 and IL-5 mRNA upon infection. Our data are in agreement with previous studies where IL-4 and IL-5 positive cells were detected at the tapeworm anchor site [33]. IL-4 is an important driver in T helper cell development leading to Th2 responses guided by antigen presenting cells [34]. Additionally, IL-5 is required for eosinophils maturation and is frequently produced along other Th2 type cytokines such as IL-4. The fact that rTsCRT did not stimulate expression of these two Th2 cytokines suggests that other parasite factors present in the TsCE besides TsCRT, contribute to the Th2 response prevailing during *T*. *solium* infection.

The golden hamster model of taeniosis reflects to some extent the early stages of infection produced by the *T*. *solium* tapeworm in humans. In this study, no proliferation in response to antigen stimuli was observed in MLN cells. Conversely, spleen cells showed a rather strong cell proliferation *in vitro* upon stimulation. During experimental taeniosis, parasite antigens are present in circulation, and this could explain, at least partially, the priming of spleen cells [18]. The lack of response in MLN, might depend on “local” dynamics involved in the immune response being elicited and may vary based on the participating cell subpopulations, as well as the prevailing cytokine microenvironment. MLN display unique immunologic properties and are programmed for tolerance induction [35]. For instance, *Trypanosoma cruzi* infection leads to lymphocyte depletion in the thymus and MLN due to apoptosis, while subcutaneous lymph nodes and spleen cells undergo vigorous lymphocyte proliferation [36,37]. Other mechanisms, such as cytokine deprivation by subsets of T cells or tolerogenic dendritic cells or macrophages might promote cell anergy and/or hyporesponsiveness, impairing local cell immunity [38,39]. Our results are in agreement with the notion that parasite infections commonly lead to different degrees of immunosuppresion [31,40]. Nonetheless, the relative unresponsiveness of immune cell in specific lymphoid organs during *T*. *soilum* infection warrants further research.

Our western blot analysis suggests that TsCRT is secreted by *T*. *solium* adult parasites. TsCRT is also present in crude extracts of cysticerci and tapeworms and its localization suggests a role in parasite development [12]. Likewise, CRT has been identified as a secreted protein in different nematodes [41]. The identification of anti-TsCRT IgG antibodies observed in one third of infected hamsters suggests that TsCRT is secreted *in vivo*.

In conclusion, this is the first study characterizing the Th2 immune response elicited by rTsCRT upon *T*. *solium* infection. The identification of helminth-derived molecules playing a role in the host-parasite interface during experimental taeniosis is an area of current study that may help unveil and better understand the host anti-parasite response. The ability of rTsCRT to induce IL-4 and IL-10 expression in cells from uninfected hamsters might be explained by its interaction with immature dendritic cells or tolerogenic macrophages. Our results suggest that the host response against rTsCRT features a robust IL-10 response. Future studies should aim to analyze the interaction of rTsCRT with different types of immune cells and elucidate the receptors and signaling pathways involved, as well as the resulting phenotypic changes of the responding cells.
