# Correction: Cytokine, Antibody and Proliferative Cellular Responses Elicited by *Taenia solium* Calreticulin upon Experimental Infection in Hamsters

**DOI:** 10.1371/journal.pone.0141177

**Published:** 2015-10-15

**Authors:** Fela Mendlovic, Mayra Cruz-Rivera, Guillermina Ávila, Gilberto Vaughan, Ana Flisser

There is a funder missing from the Funding section. The correct funding information is as follows:

Partial funding was given by CONACYT (Project 129683). AV BioSciences provided support in the form of salary for GV at the time of consultancy, but did not have any additional role in the study design, data collection and analysis, decision to publish, or preparation of the manuscript. The specific roles of this author is articulated in the ‘author contributions’ section.

Additionally, the following sentence is missing from the Acknowledgements section: Fela Mendlovic did her PhD at the National University of Mexico (Universidad Nacional Autonoma de México, UNAM) in the Biomedical Sciences Graduate Program at the Faculty of Medicine (Doctorado en Ciencias Biomédicas, Facultad de Medicina, UNAM).
